# Melanin-concentrating hormone during lactation: a compensatory role for sleep deprivation?

**DOI:** 10.3389/fnins.2026.1828432

**Published:** 2026-07-29

**Authors:** Patricia Tatemoto, Ammir Yacoub Helou, Allan dos Anjos-Monteiro, Antoine Adamantidis, Jackson Cioni Bittencourt

**Affiliations:** 1 Laboratory of Chemical Neuroanatomy, Department of Anatomy, Institute of Biomedical Sciences, Universidade de São Paulo, São Paulo, SP, Brazil; 2 Department of Neurology, Zentrum für Experimentelle Neurologie, Inselspital University Hospital Bern, Bern, Switzerland; 3 Department of Biomedical Research, University of Bern, Bern, Switzerland; 4 Center for Neuroscience and Behavior, Institute of Psychology, Universidade de São Paulo, São Paulo, Brazil

**Keywords:** hypothalamus, maternal well being, medial preoptic area (mPOA), neuroplasticity, postpartum, REM sleep

## Abstract

We propose a novel hypothesis regarding the role of melanin-concentrating hormone (MCH) in the medial preoptic area (MPOA) during lactation and its implications for mothers’ sleep patterns. We propose that MCH, with its transient expression in the MPOA during lactation, may function as a compensatory mechanism to mitigate the adverse effects of chronic sleep deprivation commonly experienced by lactating mothers. This compensatory role is hypothesized to involve regulating sleep architecture, particularly by promoting rapid eye movement (REM) sleep and modulating the balance between REM and non-rapid eye movement (NREM) sleep, which is crucial for maintaining sleep quality and recovery. The hypothesis is substantiated by key lines existing of evidence, including the adaptive sleep mechanisms observed in migratory birds and the established role of MCH in promoting REM sleep and influencing food intake following sleep deprivation. We contemplate its potential scientific and clinical implications, emphasizing the profound impact this understanding could have on improving maternal well-being and addressing sleep-related challenges during lactation. Our hypothesis sheds light on the intricate neuroendocrine interactions during a critical phase of maternal life. It opens avenues for future empirical investigations and the development of targeted interventions to enhance maternal health and sleep quality.

## Introduction

Lactating individuals frequently experience sleep deprivation (Tóth et al., 2020; Peña et al., 2023), and mechanisms to mitigate its effects may have evolved. The trade-offs of sleep deprivation may occur in other contexts, and this concept is mirrored in the adaptive behaviors of migratory birds, which exhibit evolved mechanisms allowing them to endure significant sleep reduction during migration without apparent cognitive harm (Rattenborg et al., 2004). Interestingly, these birds seem to withstand sleep loss during specific periods, suggesting that sleep quality and duration might be ‘negotiable’ during critical phases, such as migration, due to other overriding demands (Rattenborg et al., 2000; Rattenborg et al., 2004; Fuchs et al., 2006). These species of songbirds undergo nocturnal migrations, drastically reducing sleep by about two-thirds during these periods (Rattenborg et al., 2004; Fuchs et al., 2006). These birds do not fly continuously for days or weeks but rather engage in nocturnal migratory flights, typically stopping during the day to rest or forage. While nocturnal migration offers advantages, it inevitably comes at the cost of significant nighttime sleep loss during the migratory season (Fuchs et al., 2006). Evolutionary mechanisms, therefore, may have favored complementary or alternative mechanisms to compensate for sleep loss, such as unihemispheric sleep (Rattenborg et al., 2000), sleep during migratory flight, physiological resilience to sleep deprivation, or enhanced sleep quality during the limited time available for nocturnal sleep, among others.

Despite significant sleep loss during migration, the cognitive performance on a repeated acquisition task remains unaffected (Rattenborg et al., 2004). In contrast, during the nonmigratory season, even one night of experimentally induced sleep loss causes notable cognitive decline (Rattenborg et al., 2004) - and compromised cognition due to sleep deprivation, which was extensively shown across phylogenetic groups (e.g., Palchykova et al., 2006; Havekes et al., 2015; Pinheiro-da-Silva et al., 2018; Promsote et al., 2023). Another demonstrated example of behavioral adaptation to seasonal sleep loss is daytime napping observed in a species of bird (Fuchs et al., 2006). These findings suggest that migratory songbirds possess a remarkable ability to minimize sleep during migration without impairing cognitive function, showcasing a unique adaptation to the seasonal sleep deprivation (Rattenborg et al., 2004; Fuchs et al., 2006).

Sleep deprivation disrupts the functional connectivity between the amygdala and medial prefrontal cortex (mPFC), leading to amygdala hyperactivity, reduced prefrontal inhibition, and impaired emotional regulation, resulting in poor decision-making and inappropriate social judgments (Yoo et al., 2007; Khan and Al-Jahdali, 2023). Also, sleep deprivation seems to compromise memory consolidation in the hippocampus through long-term potentiation (Havekes et al., 2012; Khan and Al-Jahdali, 2023). Ultimately, the impairment in cognitive skills resulting from sleep deprivation can lead to higher vulnerability to risks and accidents, even in simple routine life activities.

It is critical to note distinct species-specific differences in sleep organization. While human NREM sleep is clinically subdivided into three stages (N1-N3), rodent sleep is most commonly dichotomized into two distinct states: NREM (characterized by high-amplitude slow waves) and REM (characterized by low-amplitude theta rhythm), without the progressive staging seen in humans (Fisher et al., 2012; Rayan et al., 2024). In rodents, NREM sleep is generally considered a single state, defined by high-amplitude, low-frequency cortical activity, without further standardized stage differentiation (Lu et al., 2000; Szymusiak et al., 2007; Monti et al., 2013). Sleep exhibits pronounced sexual dimorphism, with female mice generally showing reduced total sleep duration and distinct patterns of sleep–wake organization relative to males (Paul et al., 2006). Such differences are strongly influenced by ovarian hormones, especially estrogen, which dynamically regulate neural circuits governing arousal, circadian rhythms, and sleep homeostasis (Vida et al., 2008). Nevertheless, despite these sex-dependent variations in sleep architecture, sleep fragmentation caused by recurrent nursing demands remains a conserved physiological challenge across mammalian species during lactation.

Adaptive responses to sleep disruption during reproductive transitions are not restricted to birds. Female killer whales and bottlenose dolphins forgo sleep for weeks after giving birth, remaining continuously vigilant alongside their calves without overt behavioral signs of impairment (Lyamin et al., 2005). Northern fur seals dramatically reduce sleep during the breeding season while preserving cognitive and motor performance (Lyamin et al., 2008). These mammalian examples extend beyond the avian analogy and suggest that the brain can recruit state-specific mechanisms to modulate sleep need during evolutionarily critical life stages––a principle that motivates the hypothesis that lactating rodents may deploy a targeted neuroendocrine adaptation of their own.

Melanin-concentrating hormone (MCH), encoded by the prepro-melanin-concentrating hormone (*Pmch*) gene (Nahon et al., 1989; Nahon, 1994), is a neuropeptide predominantly expressed in neurons of the lateral hypothalamic area (LHA), the incerto-hypothalamic area (IHy), and the dorsolateral zona incerta (dlZI) (Bittencourt et al., 1992; Bittencourt, 2011). MCH neurons are maximally active during REM sleep, are preferentially recruited following sleep deprivation, and their optogenetic or pharmacological activation robustly promotes sleep, in particular REM sleep (Hassani et al., 2009; Jego et al., 2013; Konadhode et al., 2013; Verret et al., 2003; Modirrousta et al., 2005; Potter and Burgess, 2022). Critically, an additional MCH-expressing population is transiently induced in the medial preoptic area (MPOA) during lactation––first reported by Knollema et al. (1992), anatomically characterized by Rondini et al. (2010), and shown to be maintained by prolactin signaling (Kokay et al., 2020). The appearance of MCH within a primary sleep-regulatory hub, exclusively during the period of greatest maternal sleep disruption, is the neurobiological observation that motivates the present hypothesis.

Could the MCH, with its transient presence in the ventromedial part of the ventromedial preoptic area (vmMPOA) during lactation (Knollema et al., 1992; Rondini et al., 2010), serve as a compensatory mechanism against the adverse effects of sleep deprivation? Sleep is a vital, cyclic process essential for optimal organism functioning, and its deprivation is known to impair a range of functions, including immune regulation, metabolic control, and neurocognitive performance (Yoo et al., 2007; Trošt Bobić et al., 2016; Khan and Al-Jahdali, 2023). In accordance with this rationale, MCH promotes REM sleep (Hassani et al., 2009; Torterolo et al., 2011; Jego et al., 2013; Monti et al., 2013; Potter and Burgess, 2022), and evidence shows MCH neuron activation can suppress wakefulness (Konadhode et al., 2013). During lactation, sleep deprivation is a chronic condition due to the offspring’s demands (Peña et al., 2023). New mothers experience a dramatic disruption in sleep continuity. While the total sleep time may not always decrease drastically, the architecture of sleep is fundamentally altered.

The hypothesis emerges that MCH neurons might represent a compensatory mechanism to chronic sleep deprivation in lactating mothers, possibly through the emergence of MCH-expressing neurons in the vmMPOA and their projections to brain regions involved in sleep architecture. These targets may include the ventrolateral preoptic area (VLPO), dorsal raphe nucleus (DRN), locus coeruleus (LC), tuberomammillary nucleus (TMN), ventrolateral periaqueductal gray (vlPAG), suprachiasmatic nucleus (SCN), and lateral hypothalamic area (LHA), all of which are key components of the neural circuitry governing sleep–wake states and circadian regulation (Bouâouda and Jha, 2023). Notably, the MPOA maintains extensive reciprocal connectivity with both regions, positioning it as a potential integrative hub through which postpartum MCH signaling could influence sleep regulation.

We propose specifically that vmMPOA MCH neurons, activated by the homeostatic sleep pressure accumulated between nursing bouts, project to VLPO sleep-promoting neurons and/or directly suppress arousal-promoting LC and DRN neurons, thereby facilitating REM sleep onset during the fragmented sleep windows available to lactating dams. This circuit logic is consistent with the demonstration that the MCH system is required for ambient-temperature-driven REM expression and that optogenetic activation of MCH neurons extends REM bouts (Jego et al., 2013; Komagata et al., 2019).

Before delving into the functional implications, it is necessary to clarify what the lactation-induced MCH neurons in the vmMPOA represent at the cellular level. Are these neurons newly born during the postpartum period, or are they pre-existing MPOA neurons that begin expressing MCH (i.e., a phenotypic switch)? This distinction is fundamental for understanding the nature of plasticity, and to the best of our knowledge, this question has also not been elucidated. Pregnancy and lactation are known to alter the maternal brain in many ways, including changes in dendritic architecture, synaptic remodeling, and even adult neurogenesis in certain regions (Pawluski et al., 2016; Leuner and Sabihi, 2016). Some hormonal milieus of pregnancy can induce lasting changes in neuronal properties; for instance, experimental hormone treatments mimicking pregnancy can reorganize neurons in the MPOA (Kinsley et al., 2001; Ammari et al., 2023). Thus, it is plausible that existing neurons in the MPOA could be “reprogrammed” by the postpartum hormonal environment to express MCH, even if they did not do so previously. On the other hand, adult neurogenesis in the hypothalamus is a less explored phenomenon, but not inconceivable given the extensive plasticity observed in the maternal brain.

Resolving this question will require a combination of molecular, anatomical, and functional approaches. Single-cell transcriptomic profiling across gestation, lactation, and post-weaning could determine whether an MCH-expression program emerges *de novo* within pre-existing MPOA neuronal populations or reflects state-dependent transcriptional remodeling. Complementary hormonal manipulation studies, including prolactin receptor conditional knockout models, hyperprolactinemia paradigms, and manipulations of gonadal steroid signaling, would help identify the endocrine mechanisms driving this plasticity. In parallel, anterograde tracing from vmMPOA MCH neurons could define their downstream connectivity within sleep- and maternal behavior-related circuits.

Importantly, functional studies will be essential to establish the behavioral relevance of these neurons. Optogenetic or chemogenetic manipulation of vmMPOA MCH neurons combined with sleep analyses and maternal behavior assays could directly test their contribution to sleep regulation and caregiving during lactation. Fiber photometry recordings would further allow monitoring of vmMPOA MCH neuronal activity across sleep–wake states and during nursing, pup-directed interactions, and other maternal behaviors. Coupling these approaches with simultaneous EEG/EMG recordings would enable direct correlations between neuronal activity and specific vigilance states, providing mechanistic insight into how these cells influence sleep architecture in the postpartum period.

Although lineage-tracing strategies may provide valuable information regarding the developmental origin of these neurons, the availability of established *Pmch*-Cre mouse lines offers an immediate and powerful framework for selectively targeting MCH-expressing cells. Together, these approaches would help determine whether the emergence of MCH neurons in the vmMPOA represents a transient neuroendocrine adaptation linking maternal behavior, hormonal state, and sleep homeostasis during lactation.

The MPOA plays a pivotal role in lactation and other physiological processes. However, the intricate interplay between MCH and the vmMPOA, especially in lactation, has not been fully elucidated. This manuscript introduces a comprehensive hypothesis that aims to bridge this gap, exploring the potential role of MCH in modulating sleep patterns during lactation. MCH neurons in the vmMPOA may significantly influence sleep by modulating maternal behavior (Benedetto et al., 2018) and potentially influencing sleep through MCH-mediated circuits (Ferreira et al., 2017). MCH expression has been documented to peak on the 19th day of lactation exclusively within the vmMPOA, suggesting a potential link between MCH and the cessation of maternal behavior (Knollema et al., 1992; Rondini et al., 2010; Li, 2024). This state is correlated with increased sleep and synchronized electroencephalogram (EEG) activity preceding milk ejection (Lincoln et al., 1980). Moreover, variations in REM sleep duration in female rats during proestrus nights suggest an inhibitory role of MCH neurons, an observation demanding further exploration of lactation and different reproductive states (Ferreira et al., 2017).

While the broader preoptic area is a canonical site for sleep-active neurons that inhibit arousal systems (Saper et al., 2010), this region is composed of highly heterogeneous and molecularly distinct neuronal populations. Within this complex landscape, the vmMPOA is uniquely positioned to integrate homeostatic drive with sleep–wake transitions. The appearance of MCH within this specific homeostatic center during lactation suggests a mechanism to bypass standard circadian constraints, allowing the dam to maximize restorative sleep (specifically REM) during the fragmented intervals available between nursing bouts.

## MCH in the vmMPOA as a buffer against sleep deprivation in lactating mothers

MCH plays a key role in the intricate orchestration of biological functions related to sleep, lactation, and neuroplasticity. MCH neurons are predominantly active during REMv sleep and (Hassani et al., 2009; Potter and Burgess, 2022). Their activity increases following sleep deprivation MCH neuronal activity suggesting a potential compensatory or homeostatic role in sleep regulation (Bandaru et al., 2020; Verret et al., 2003; Modirrousta et al., 2005). In line with this role, sleep deprivation triggers REM rebound, a compensatory increase in REM sleep during recovery periods (Beersma et al., 1990). Furthermore, MCH signaling contributes to the stabilization and maintenance of REM sleep episodes (Potter and Burgess, 2022).

Sleep deprivation poses a serious threat to brain function, impacting learning, memory, and synaptic plasticity (Havekes et al., 2012; Areal et al., 2017). The alteration in the state of cognition-related signaling molecules due to lack of sleep underscores sleep’s vital role in maintaining cognitive integrity (Havekes et al., 2012; Trošt Bobić et al., 2016; Khan and Al-Jahdali, 2023). The interplay between sleep, neural plasticity, and cognitive function is nuanced. It varies with age, individual differences, and physiological contexts––such as lactation, further complicating the landscape of sleep-related neurobiological consequences (Yoo et al., 2007; Havekes et al., 2012; Trošt Bobić et al., 2016; Khan and Al-Jahdali, 2023).

The role of MCH in memory regulation is nuanced and likely context-dependent (Chee et al., 2015; Izawa et al., 2019). It has been shown that MCH neurons co-expressing cocaine- and amphetamine-regulated transcript (CART) preferentially project to the hippocampus and exhibit specific activity during REM sleep (Cvetkovic et al., 2004). Notably, this MCH-CART hippocampal projection has been linked to mechanisms of ‘active amnesia’ during sleep, facilitating the erasure of irrelevant information (Izawa et al., 2019).

**Figure 1:**
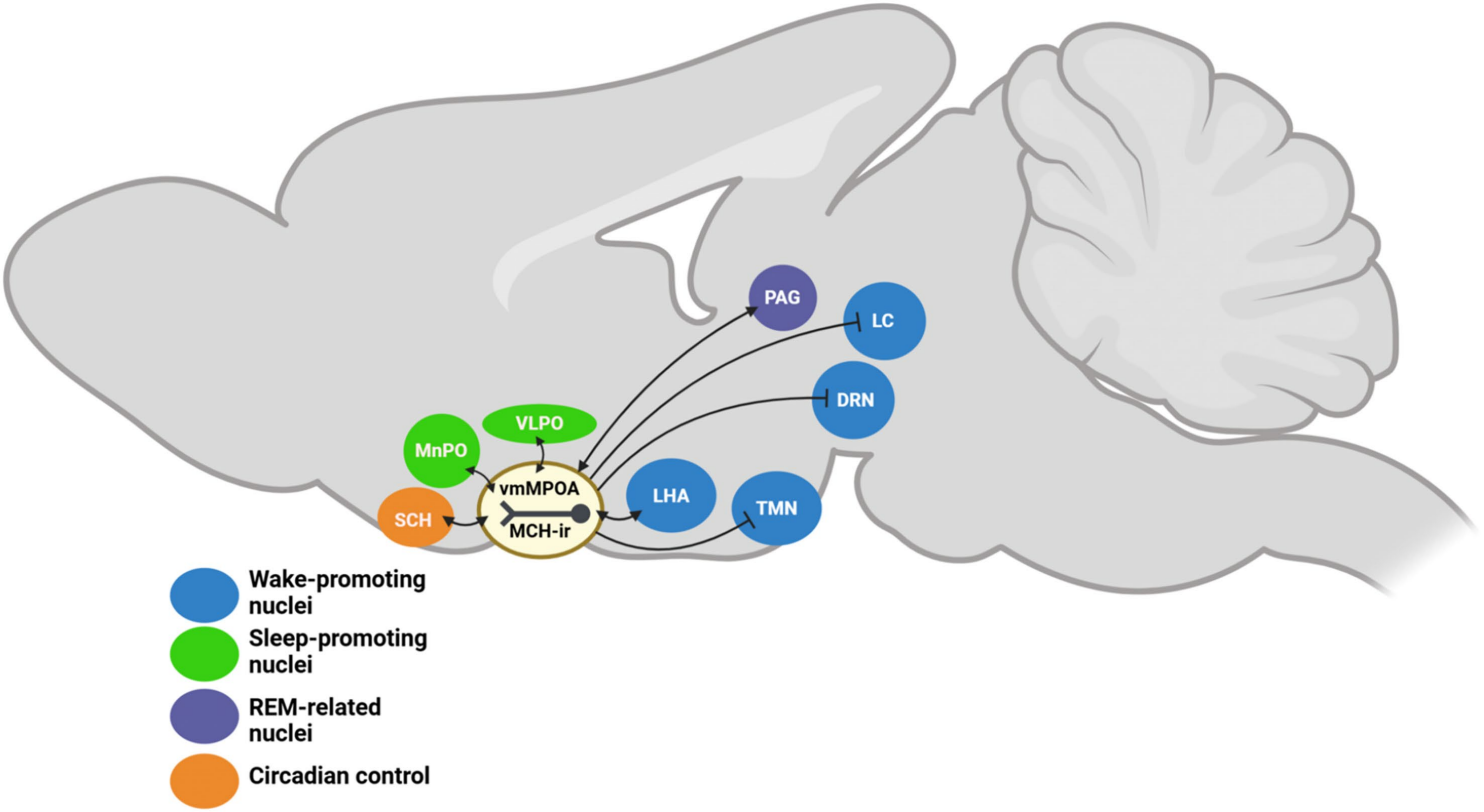
Proposed interactions between vmMPOA MCH-immunoreactive neurons and neural circuits regulating sleep–wake states during lactation-associated sleep deprivation.

However, we propose that the transient recruitment of MCH neurons in the vmMPOA during lactation serves a distinct physiological mandate. Unlike the LHA-derived projections associated with hippocampal forgetting, the vmMPOA population is induced by the high homeostatic demand of nursing and likely projects to arousal-regulatory centers (e.g., LC/DRN) rather than primarily to the hippocampus. Thus, we suggest that while LHA-MCH signaling may participate in memory gating, the lactation-induced vmMPOA-MCH circuit acts primarily as a homeostatic rheostat, buffering against the global cognitive impairments associated with chronic sleep fragmentation (Figure 1).

MCH-ir neurons located in the vmMPOA are positioned to possibly influence multiple components of the sleep–wake regulatory network. Putative interactions are illustrated with sleep-promoting nuclei (green; VLPO, ventrolateral preoptic nucleus; MnPO, median preoptic nucleus), wake-promoting nuclei (blue; LHA, lateral hypothalamic area; TMN, tuberomammillary nucleus; LC, locus coeruleus; DRN, dorsal raphe nucleus), REM sleep-related structures (purple; PAG, periaqueductal gray), and the circadian pacemaker (orange; SCN, suprachiasmatic nucleus). Arrows indicate putative inhibitory-excitatory or facilitatory influences, whereas blunt-ended lines indicate inhibitory influences. This schematic summarizes experimentally supported and hypothesized pathways through which vmMPOA MCH neurons may contribute to the regulation of sleep, wakefulness, and REM sleep.

**Figure 2:**
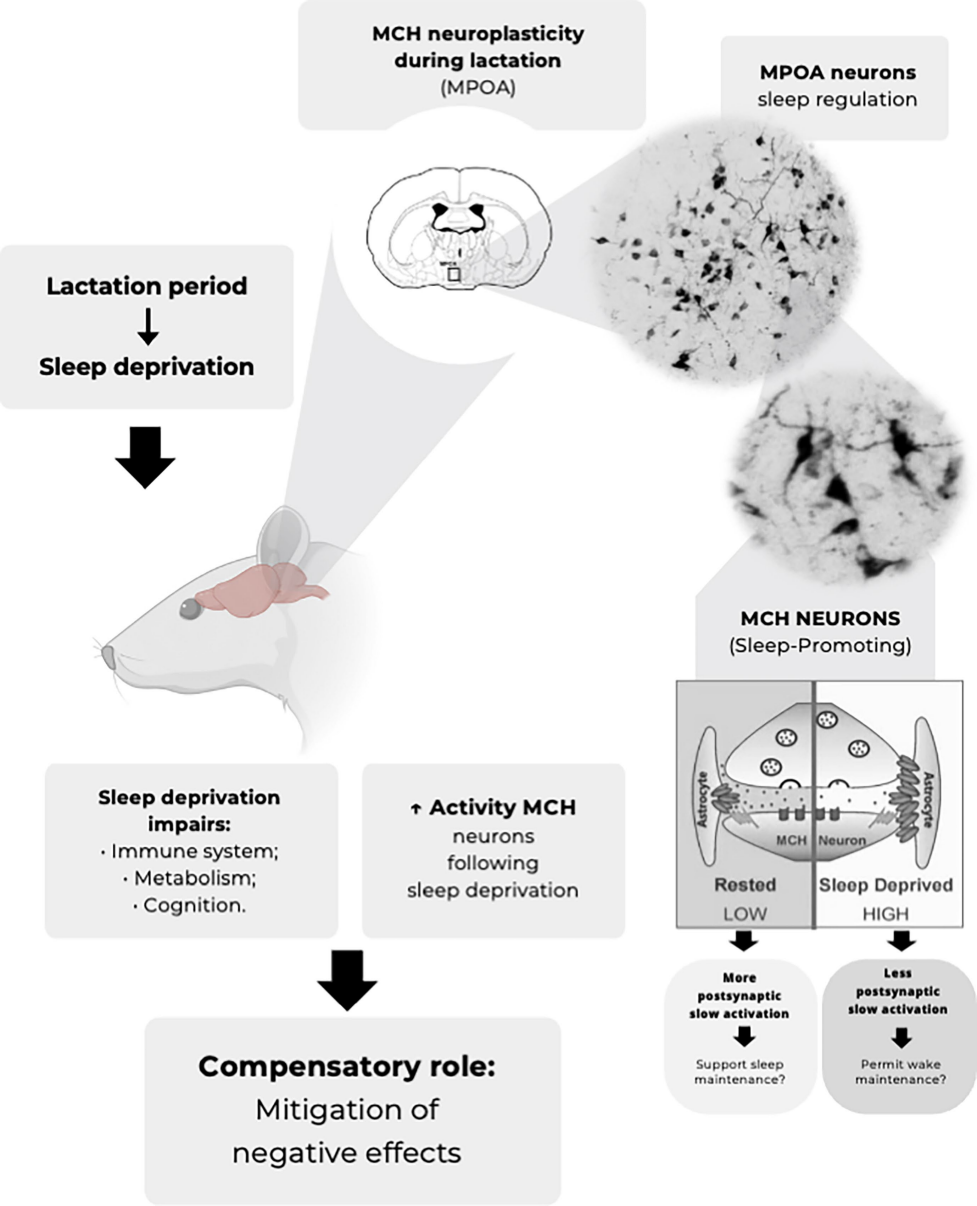
Schematic representation of the proposed hypothesis, focusing on the neuroplastic emergence of MCH-expressing neurons specifically in the vmMPOA during lactation. The lactation period typically involves substantial maternal sleep deprivation, impairing immune function, metabolic homeostasis, and cognitive performance. The transient induction of MCH expression within vmMPOA neurons is hypothesized as an adaptive neuroendocrine response aiming to mitigate these adverse effects. Highlighted are the neuronal mechanisms through which MCH signaling might exert protective functions, potentially promoting restorative REM sleep and stabilizing sleep–wake cycles disrupted during maternal caregiving. The increased activity of MCH neurons under sleep deprivation conditions may influence synaptic signaling pathways, facilitating compensatory adjustments to maintain maternal health and behavior. The bottom right panel illustrates synaptic mechanisms potentially underlying these adaptations, depicting altered astrocytic function and changes in metabotropic glutamate receptor (mGluR) activity, resulting in differential postsynaptic slow activation between rested and sleep-deprived states (adapted from Briggs et al., 2018). Such synaptic plasticity may contribute to maintaining an optimal balance between sleep and wakefulness during the lactation period.

In advancing this hypothesis (Figure 2), we explicitly recognize that several other systems are already implicated in maternal sleep regulation, and that vmMPOA MCH is unlikely to operate in isolation. Prolactin itself promotes slow-wave/NREM sleep in breastfeeding women (Blyton et al., 2002) and is required to maintain vmMPOA MCH expression (Kokay et al., 2020), positioning vmMPOA MCH as one downstream effector of prolactin-driven sleep adaptation rather than a parallel alternative. Drawing on evidence that intra-MPOA MCH infusion reduces active maternal behaviors (Benedetto et al., 2018), it has recently been proposed that the progressive rise of vmMPOA MCH across late lactation is the neurochemical signal that drives the natural decline of active maternal care, which acts through inhibitory projections to mesolimbic dopamine systems and excitatory projections to ventral-tegmental serotonergic systems (Li, 2024). We regard this view and ours as complementary rather than mutually exclusive: vmMPOA MCH may simultaneously dampen active maternal-approach motivation (the Li/Benedetto framework) and improve REM-sleep efficiency under fragmentation (the framework proposed here). Both effects are downstream of prolactin, both peak around postpartum day 19, and both are addressable by the chemogenetic-inhibition experiment (Prediction 2 below) — which would be expected to attenuate both maternal-care decline and the proposed sleep buffering, providing a single experimental adjudication between the two views.

As outlined previously, sleep is an important factor for the well-being of organisms and has been extensively investigated. In some periods of life, there is an increased vulnerability, such as the gestation and lactation periods. Proper sleep is suggested to be involved in the reduction of the incidence of postnatal depression (Iranpour et al., 2016; Okun et al., 2018), and there is some clinical evidence that sleep appears to play an important role in maintaining milk supply (Blyton et al., 2002). A correlation has been reported between slow-wave sleep (SWS) duration and milk production, emphasizing the need for preserved sleep, especially SWS sleep, during breastfeeding (Aerts et al., 2023). Nevertheless, while alterations in sleep architecture have been described in breastfeeding women (Blyton et al., 2002), it remains unknown whether MCH expression is induced during lactation in humans. The most likely explanation for the altered sleep architecture observed in women who are exclusively breastfeeding their infants is the increase in circulating prolactin levels that occurs during lactation (Blyton et al., 2002), and there is a direct relationship between prolactin secretion and MCH production (Kokay et al., 2020). Thus, understanding and supporting proper sleep during lactation is crucial not only for maternal mental health but also for optimizing breastfeeding outcomes, which in turn has an important adaptive role.

## vmMPOA Pmch neurons mediate sleep homeostasis during lactation

**Figure 3:**
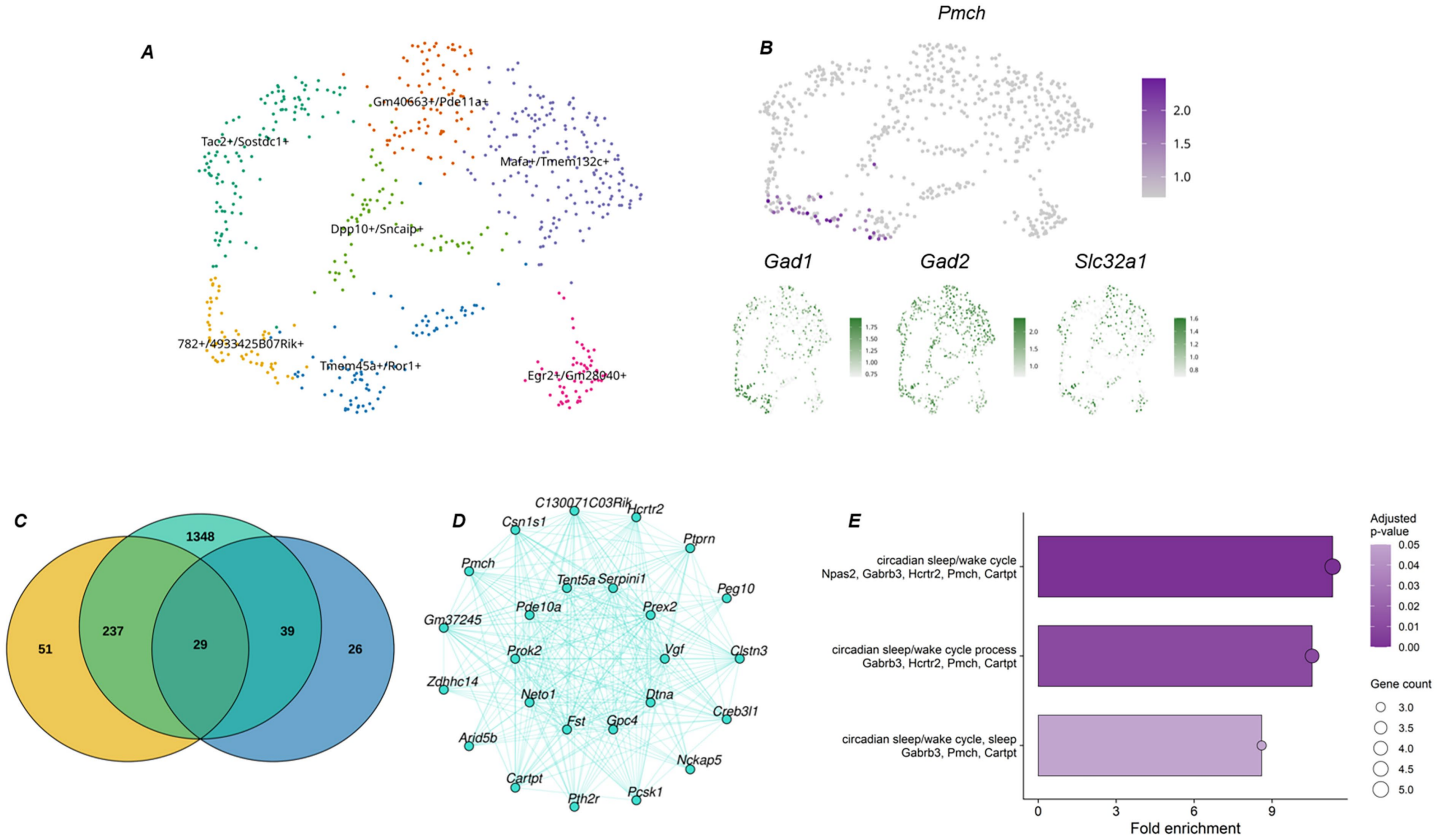
Molecular and functional characterization of MPOA Pmch neurons reveals their engagement in sleep–wake regulation. **(A)** UMAP projection of MPOA single-cell transcriptomic data showing the organization of neuronal subpopulations with distinct clusters defined by canonical marker genes, in which *Pmch*-expressing cells are embedded within transcriptionally heterogeneous neuronal groups, suggesting functional specialization. **(B)** Feature plots illustrating the spatial distribution of *Pmch* alongside GABAergic markers *Gad1*, *Gad2* and *Slc32a1*, indicating that MPOA *Pmch* neurons are predominantly inhibitory and therefore well positioned to modulate circuit excitability across sleep and wake states. **(C)** Venn diagram depicting the overlap of gene sets across conditions, highlighting a shared core of genes potentially involved in circadian and homeostatic regulation. **(D)** Co-expression interaction network of *Pmch*-associated genes revealing a highly interconnected module including *Npas2*, *Hcrtr2*, *Gabrb3*, *Cartpt* and *Pmch*, consistent with convergence of circadian transcriptional programs, inhibitory neurotransmission and neuropeptidergic signaling. **(E)** Gene Ontology enrichment analysis identifying significant overrepresentation of sleep–wake-related processes including circadian sleep/wake cycle terms, driven by genes such as *Npas2*, *Gabrb3*, *Hcrtr2*, *Pmch* and *Cartpt*, which collectively support a model in which MPOA *Pmch* neurons integrate circadian timing, GABAergic inhibition, orexin-dependent arousal signaling and neuropeptidergic modulation of behavioral states. Together, these data indicate that MPOA *Pmch* neurons constitute a transcriptionally defined inhibitory hub linking circadian and neuromodulatory pathways to the regulation of sleep–wake architecture, with potential relevance for state transitions during physiologically demanding conditions such as lactation.

Providing molecular support for our circuit-level model, our recent single-cell RNA-sequencing analysis identified two transcriptionally distinct populations of transient *Pmch*-expressing neurons within the vmMPOA of lactating mice dams, namely a *Pmch/Iapp* population and a *Pmch/Penk/Pdyn* population, representing, to our knowledge, the first description of *Pmch* expression in this region during lactation using sc-RNA-seq (Anjos-Monteiro et al., 2026). Although these populations exhibit distinct molecular identities, both emerge selectively during lactation and display transcriptional features suggestive of involvement in sleep-related and homeostatic processes (Figures 3A–D).

Gene ontology enrichment analysis revealed significant overrepresentation of biological processes associated with circadian sleep (GO:0042745), circadian sleep/wake cycle process (GO:0022410), and circadian sleep/wake cycle, sleep (GO:0050802) (Figure 3E). These enrichments were driven by genes including neuronal PAS domain protein 2 (*Npas2*), gamma-aminobutyric acid type A receptor subunit beta-3 (*Gabrb3*), hypocretin receptor 2 (*Hcrtr2*), solute carrier family 32 member 1 (*Slc32a1*), *Pmch*, and cocaine- and amphetamine-regulated transcript prepropeptide (*Cartpt*), suggesting the existence of a coordinated molecular program linking circadian timing, sleep regulation, and behavioral state control.

The expression of *Npas2* suggests coupling to endogenous circadian timing mechanisms, whereas the expression of glutamate decarboxylase 1 (*Gad1*), glutamate decarboxylase 2 (*Gad2*), *Slc32a1*, and *Gabrb3* supports a GABAergic phenotype consistent with the established role of preoptic inhibitory circuits in suppressing arousal-promoting systems during sleep (Figure 2B). Particularly intriguing is the expression of *Hcrtr2*, which places these neurons within a molecular framework capable of interacting directly with orexin-dependent arousal networks. Together with the expression of *Pmch* and *Cartpt*, these findings suggest the integration of circadian, inhibitory, metabolic, and arousal-related signaling pathways.

Taken together, these data support the existence of a lactation-induced transcriptional program within vmMPOA *Pmch* neurons enriched for genes associated with circadian regulation, inhibitory neurotransmission, and sleep–wake control. Rather than representing a simple induction of *Pmch* expression, these findings suggest the emergence of specialized neuronal states during lactation. While these observations do not establish a direct role in sleep regulation, they provide molecular evidence consistent with the hypothesis that transient vmMPOA *Pmch* populations contribute to adaptive responses to the fragmented sleep and increased physiological demands of lactation (Figures 3A–E).

## Testable predictions

The hypothesis generates the following concrete, falsifiable predictions:

1. Optogenetic activation of vmMPOA MCH neurons in lactating dams during inter-nursing intervals should increase REM sleep bout frequency and duration, quantified by EEG/EMG polysomnography.
2. Chemogenetic inhibition (e.g., hM4Di DREADD) of vmMPOA MCH neurons in lactating rats should worsen performance on hippocampus-dependent memory tasks and on object recognition, relative to lactating controls and to non-lactating inhibited animals.
3. Genetic fate-mapping (Cre-lox or intersectional Dre-rox strategies with an inducible MCH-Cre line) should determine whether vmMPOA MCH neurons represent newly born cells or a phenotypic switch of pre-existing MPOA neurons.
4. Single-cell RNA sequencing of vmMPOA neurons across gestation (days 1, 10, 19) and after weaning should reveal a temporally regulated transcriptional program including Pmch induction peaking near day 19.
5. Anterograde tract-tracing from vmMPOA MCH neurons (e.g., AAV-Cre-dependent synaptophysin-GFP in MCH-Cre mice during lactation) should map their projection targets onto established sleep-regulatory nuclei, including VLPO, DRN, LC, SLD, and vlPAG/LPT.

Each of these predictions is addressable with existing reagents and can, in principle, falsify the hypothesis.

## Conclusion

Here, we proposed a novel hypothesis that MCH might play a compensatory role during lactation, mitigating the adverse effects of chronic sleep deprivation commonly experienced in mammal mothers. This hypothesis is supported by the evidence from adaptive sleep mechanisms in migratory birds and the established role of MCH in promoting REM sleep. Importantly, the comparison between migratory birds and lactating mammals should be viewed as an evolutionary and behavioral heuristic rather than evidence of a shared neurobiological mechanism. Nevertheless, it highlights the capacity of organisms to transiently override sleep pressure when competing survival demands, such as migration or offspring care, take precedence. This framework opens new avenues for research and targeted interventions to enhance maternal health and quality of life.

If validated in future preclinical and, eventually, clinical studies, the MCH-MPOA-sleep interaction may suggest novel neuroendocrine targets for supporting maternal sleep. Significant translational gaps remain, most notably the absence of direct evidence for MCH induction during human lactation, and any clinical inference must be regarded as aspirational until such evidence is established.

It is important to acknowledge that while the presence of MCH-expressing neurons in the MPOA during lactation has been well documented, their specific projection targets and electrophysiological properties remain largely unknown. Notably, recent single-cell transcriptomic analyses identified two transients *Pmch*-expressing neuronal populations in the vmMPOA of lactating mice, providing initial molecular support for the hypothesis proposed here. Future studies combining circuit mapping, functional manipulations, and *in vivo* recordings will be required to determine whether these neurons are integrated into sleep-regulatory networks or primarily contribute to other lactation-associated adaptations.

These insights delineate a nuanced interplay between MCH expression in the MPOA and lactation, contributing to the growing understanding of the neuroendocrine control of reproductive behaviors and physiological adaptations during this period. The characterization and hormonal regulation of MCH-expressing neurons in the MPOA underscore the potential intricacies in elucidating the role of MCH in sleep regulation during lactation.

## Data Availability

The original contributions presented in the study are included in the article/supplementary material, further inquiries can be directed to the corresponding author.
